# Ovarian reserve in adolescent girls with autoimmune thyroiditis

**DOI:** 10.20945/2359-3997000000597

**Published:** 2023-02-07

**Authors:** Kotb A. Metwalley, Hekma S. Farghaly, Deiaaeldin M. Tamer, Ahmed M. Ali, Mostafa Embaby, Islam F. Elnakeeb, Eman B. Kamaleldeen

**Affiliations:** 1 Assiut University Faculty of Medicine Department of Pediatrics Assiut Egypt Department of Pediatrics, Faculty of Medicine, Assiut University, Assiut, Egypt; 2 Aswan University Faculty of Medicine Department of Clinical Pathology Aswan Egypt Department of Clinical Pathology, Faculty of Medicine, Aswan University, Aswan, Egypt

**Keywords:** Autoimmune thyroiditis, ovarian reserve, anti-Müllerian hormone, adolescent

## Abstract

**Objectives:**

To assess serum anti-Müllerian hormone (AMH) levels as an ovarian reserve marker in adolescent girls with autoimmune thyroiditis (AIT) and explore the relationship of this marker with autoimmunity and thyroid function biomarkers.

**Subjects and methods:**

This study included 96 adolescent girls with newly diagnosed AIT and 96 healthy, age- and sex-matched controls. All participants were evaluated with detailed history taking and physical examination, thyroid ultrasound, and measurement of levels of thyroid-stimulating hormone (TSH), free thyroxin (FT4), free triiodothyronine (FT3), antithyroid peroxidase antibodies (TPOAb), antithyroglobulin antibody (TGAb), estradiol, total testosterone, and anti-Müllerian hormone (AMH) levels. The LH/FSH ratio was also calculated. Among 96 patients evaluated, 78 were overtly hypothyroid and 18 were euthyroid. AMH levels were significantly lower in participants with overt hypothyroidism and euthyroidism compared with controls.

**Results:**

Serum levels of AMH correlated negatively with age, body mass index (BMI) standard deviation score (SDS), and TPOAb, TGAb, and TSH levels but positively with FT4 levels. In multivariate analysis, AMH levels correlated significantly with age (odds ratio [OR] = 1.65, 95% confidence interval [CI] 1.18-2.32, p = 0.05), BMI SDS (OR = 2.3, 95% CI, 2.23-3.50, p = 0.01), TSH (OR = 2.43, 95% CI 1.5-2.8, p = 0.01), and TPOAb (OR = 4.1, 95% CI 3.26-8.75, p = 0.001).

**Conclusions:**

Ovarian reserve of adolescent girls with AIT, as measured by serum AMH levels, is affected by thyroid autoimmunity and hypothyroidism, indicating a possible need for ovarian reserve monitoring in these patients.

## INTRODUCTION

Autoimmune thyroiditis (AIT) is the most common cause of acquired hypothyroidism in adolescents (1). Susceptible individuals prone to develop AIT are those with abnormalities in cellular immune response, antithyroid autoantibodies, immune susceptibility genes, and exposure to environmental triggers (2). Thyroid hormones play a significant role in the complex events leading to a successful pregnancy, including folliculogenesis, spermatogenesis, fertilization, embryo development, implantation, and placentation (3). Therefore, thyroid dysfunction and autoimmunity are associated with adverse effects on pregnancy and fertility. Development of AIT can occur alone or in association with other autoimmune diseases, such as type 1 diabetes mellitus, Addison's disease, and premature ovarian failure (POF) (4). The definition of POF includes the occurrence of gonadal failure before the age of 40 years, as established by clinical and laboratory findings. Abnormalities of cellular immunity and autoimmune processes have a role in the autoimmune etiology of POF, while AIT is the most common disease accompanying POF in adult women (5-7).

Anti-Müllerian hormone (AMH) is a dimeric glycoprotein that belongs to the transforming growth factor beta superfamily (8). This hormone is produced by the granulosa cells of growing ovarian follicles from fetal life to menopause, and its serum levels correlate with a low antral follicle count (9). The AMH levels remain relatively stable during the menstrual cycle and are not affected by hormone feedback mechanisms (10). Thus, AMH is a reliable marker for quantitatively evaluating ovarian reserve (3,4,8,9) due to its level remaining relatively stable during the menstrual cycle and not being affected by hormonal feedback mechanisms (3,4).

Only three studies to date have evaluated AMH levels in adolescent girls with AIT, with some showing controversial results (5-7). Based on these considerations, the aim of this study was to assess serum AMH levels as an ovarian reserve marker in adolescent girls with AIT and explore the relationship of AMH with biomarkers of autoimmunity and thyroid function. Our hypothesis was that the autoimmunity in girls with AIT would predispose them to premature ovarian insufficiency.

## SUBJECTS AND METHODS

### Patients

This cross-sectional study included 96 adolescent girls with treatment-naïve, newly diagnosed AIT. The participants were recruited as they attended the endocrinology clinic at the Children's Hospital of the Assiut University and the outpatient pediatric clinic at the Aswan University Hospital (both in Egypt). Enrollment in the study was confirmed after demonstration of elevated serum levels of antithyroid peroxidase antibodies (TPOAbs) and antithyroglobulin antibodies (TGAbs), and the typical finding of a hypoechogenic thyroid on high-resolution ultrasound (11,12). At the time of the study, the patients were either euthyroid (TSH and FT4 levels within the normal range) or overtly hypothyroid (TSH ≥ 10 mIU/L and low FT4). The participants were excluded from the study if presenting with significant illness, chronic inflammatory/autoimmune disease other than AIT, subclinical AIT (which occurred in two cases), Graves’ disease, irregular menstrual cycles, obesity, genetic syndromes, or polycystic ovary or if within the previous 6 months they used any medication known to affect the thyroid or ovarian function. They were also excluded if they were a product of a pregnancy complicated by gestational diabetes or preterm delivery or if they were born small for gestational age or from a multiple pregnancy. All adolescents in the study had a regular menstrual cycle without using oral contraceptives (the mean duration of their menstrual cycle was 28.2 ± 2.2 days, counted from the first day of the period to the day before the next period).

Serving as a control group, the study also included 96 healthy adolescent girls with a distribution of age, sex, and socioeconomic status similar to those in the AIT group. The participants with AIT were recruited from the General Pediatric Outpatient Clinic of the Assiut University Children's Hospital and Aswan University Hospital (both in Egypt). The participants in the control group were attending the outpatient clinic either because they had a minor illness or were accompanying a sick sibling. The inclusion criteria for the control group were confirmed normal serum TSH and FT4 levels, negative antithyroid antibodies, and absence of history of thyroid disease.

### Methods

All participants underwent a thorough history taking and complete physical examination specifically checking for signs of thyroid dysfunction or disease. Anthropometric measurements (height and weight) were recorded. Body mass index (BMI) was calculated as weight (in kg) divided by the squared height (in m^2^) and was expressed as standard deviation scores (SDSs) to normalize for age and sex using the Egyptian Growth Reference Data (13). Pubertal development was assessed according to Tanner stage (14).

The protocol of the study was approved by the ethics committee of the Children's Hospital and Faculty of Medicine at the Assiut University. The study was performed according to the standards of the 1964 Declaration of Helsinki and its later amendments. Written informed consent was obtained from the legal guardians of the participants before study enrollment.

### Laboratory investigation

Early morning blood samples were obtained from all participants during the follicular phase (3-5 days) for measurement of serum levels of AMH, FSH, LH, estradiol, and total testosterone. Levels of FSH, LH, estradiol, and total testosterone were measured using chemiluminescent microparticle immunoassay (paramagnetic particle, chemiluminescent immunoassay; Unicel DxI 800 System, Beckman Coulter Inc., Brea, CA, USA) with original reagents. Levels of AMH were measured using an enzyme-linked immunosorbent assay (ELISA) kit (Cusabio, Houston, TX, USA). Levels of TSH, FT4, and FT3 were measured using ultrasensitive immunometric assays (IMMULITE 2000 Third Generation, Diagnostic Products Corporation, Los Angeles, CA, USA). The reference range for the thyroid hormones was as follows: TSH = 0.4-4.0 mIU/L, FT3 = 3.5-5.5 pmol/L, and FT4 = 10.0-26.0 pmol/L. Serum TPOAb and TGAb were measured using rapid ELISA (Genesis Diagnostics, Littleport, UK). Levels of TPOAb and TGAb were considered positive when greater than 100 IU/mL and 75 IU/mL, respectively, and the diagnosis of AIT was confirmed with at least one positive antibody. Overt hypothyroidism was defined as an elevated TSH level (≥10 mIU/L) plus a low FT4 level. Euthyroidism was defined as TSH and FT4 levels within the normal reference (15).

### Statistical analysis

Statistical analyses were performed using SPSS, v18.0 (IBM Corp., Armonk, NY, USA). The normality of data distribution was assessed using the Kolmogorov-Smirnov test. Normally distributed continuous variables are presented as mean ± standard deviation, and non-normally distributed variables are presented as median (range). Between-group differences were detected using Student's *t* test for parametric data and the Mann-Whitney U test for nonparametric data. Linear associations between AMH levels and other parameters were assessed using Pearson's and Spearman's correlation coefficients for normally and non-normally distributed data, respectively. Multiple logistic regression analysis (expressed as odds ratios [ORs] and 95% confidence intervals [CIs]) was used to determine significant independent associations between AMH and demographic, clinical, and laboratory variables. For all tests, values of p < 0.05 were considered statistically significant.

## RESULTS

Table 1 shows the most relevant characteristics of the participants in the AIT and control groups. All participants and controls were rated Tanner stage 5. Among the 96 participants, 78 were overtly hypothyroid and 18 were euthyroid. Patients in the AIT group (with overt hypothyroidism and euthyroidism), compared with controls, had significantly higher levels of TPOAb and TGAb and significantly lower levels of AMH (Table 1). Serum AMH levels correlated negatively with age (r = −435, p = 0.01), BMI SDS (r = −654, p = 0.001), TSH (r = −769, p = 0.001), TPOAb (r = −0.353, p = 0.01), and TGAb (r = −0.293, p = 0.05), and positively with FT4 (r = 562, p = 0.001) (Table 2). In multivariate analysis, AMH levels correlated significantly with age (OR = 1.65, 95% CI 1.18-2.32, p = 0.05), BMI SDS (OR = 2.3, 95% CI 2.23-3.50, p = 0.01), TSH (OR = 2.43, 95% CI 1.5-2.8, p = 0.001), and TPOAb (OR = 4.1, 95% CI 3.26-8.75) (Table 3).

**Table 1:** Demographic, anthropometric, and laboratory data of patients with overt hypothyroidism, euthyroidism, and controls

	Overt hypothyroidism (n = 78)	Euthyroidism (n = 18)	Controls (n = 96)	P values
Age (years)	16. 21 ± 3.32	14.10 ± 2.53	14.9 ± 1.3	0.05
BMI SDS	1.02 ± 0.81	0.96 ± 0.62	0.54 ± 1.33	0.001
TSH (µIU/mL)	79.23 ± 9.55	4.12 ± 1.20	3.10 ± 1.12	0.001
Free T4 (pmol/L)	5.21 ± 2.32	20.10 ± 2.45	22.34 ± 2.76	0.001
FT3 (pmol/L)	1.6 ± 1.1	4.3 ± 1.3	4.2 ± 2.1	0.001
LH/FSH (mIU/mL)	1.46 ± 1.2	1.52 ± 0.8	1.61 ± 0.9	0.001
Estradiol (pg/mL)	83.3 ± 34.3	85.7 ± 39.2	88.2 ± 23.3	NS
Total testosterone (ng/dL)	34.5 ± 15.2	31.7 ± 18.2	29.3 ± 14.3	NS
TGAb (IU/mL)	309.8 ± 33.1	154.4 ± 19.8	18.1 ± 8.6	0.000
TPOAb (IU/mL)	543.6 ± 72.7	245 ± 32.9	15.7 ± 3.5	0.000
AMH (ng/mL)	1.92 ± 1.07	2.71 ± 1.2	4.2 ± 1.27	0.000

Data are expressed as mean ± standard deviation.

BMI SDS: body mass index standard deviation score; TSH: thyroid-stimulating hormone; FT4: free thyroxine; FT3: free triiodothyronine; FSH: follicle-stimulating hormone; LH: luteinizing hormone; TPOAb: antithyroid peroxidase antibody; TGAB: antithyroglobulin antibody; AMH: anti-Müllerian hormone; NS: nonsignificant.

**Table 2:** Correlations between anti-Müllerian hormone (AMH) and various confounding variables in the autoimmune thyroiditis (AIT) group

Confounding variables	r	P values
Age (years)	-0.435	0.01
BMI SDS	-0.654	0.001
TSH (µIU/mL)	-0.769	0.001
FT4 (pmol/L)	0.562	0.01
TPOAb (IU/mL)	-0.353	0.01
TGAb (IU/mL)	-0.293	0.05
LH/FSH	0.104	NS
Estradiol (pg/mL)	0.136	NS
Testosterone (ng/mL)	0.101	NS

BMI SDS: body mass index standard deviation score; FSH: follicle-stimulating hormone; LH: luteinizing hormone; TSH: thyroid-stimulating hormone; FT4: free thyroxine; TPOAb: antithyroid peroxidase antibody; TGAB: antithyroglobulin antibody; NS: nonsignificant.

**Table 3:** Multivariate logistic regression models between anti-Müllerian hormone (AMH) and various confounding variables in adolescent girls with autoimmune thyroiditis (AIT)

Confounding variables	Odds ratio	95% confidence interval (CI)
Age (years)	1.65*	1.18-2.32
BMI SDS	2.35*	2.23-3.50
TPOAb ((IU/mL)	4.1***	3.26-8.75
TSH (µIU/mL)	2.43**	1.5-2.8

BMI SDS: body mass index standard deviation score; TPOAb: antithyroid peroxidase antibody; TSH: thyroid-stimulating hormone.

* Significant at p < 0.05.

** Significant at p = 0.01.

*** Significant at p = 0.001.

## DISCUSSION

In this study, AMH levels were significantly lower in patients with overt hypothyroidism and euthyroidism when compared with controls.

These results imply that the ovarian reserve, measured by serum AMH levels, was affected in children and adolescents with AIT. In line with our results, Özalp Akin and Aycan have reported lower AMH in a cohort of adolescents with euthyroid AIT (on treatment) compared with a control group (1.7 ng/mL versus 1.8 ng/mL, respectively), although this difference was not significant (p = 0.784) (5). Saglam and cols. reported that AMH levels were lower in women with AIT (n = 85) than controls (n = 80), all of whom were younger than 40 years (1.16 ± 0.17 ng/mL versus 1.28 ± 0.25 ng/mL, respectively, p = 0.001) (16). Kucukler and cols. analyzed the ovarian reserve in 42 women with AIT aged 20-40 years and reported a significant difference in AMH levels between women with subclinical hypothyroidism, overt hypothyroidism, and controls (p = 0.19) (17). Although the AMH values were not significantly different between the groups, they were lower in patients with overt hypothyroidism and subclinical hypothyroidism, prompting the authors to recommend follow-up of ovarian reserve in women with AIT. In contrast, Özalp Akin and Aycan reported that the ovarian reserve of adolescent girls, as measured by serum AMH levels, is not affected by AIT (5).

Erol and cols. (6) and Pirgon and cols. (7) reported significantly higher AMH levels in euthyroid adolescents with AIT compared with age-matched healthy controls. Studies focused on the ovarian reserve of adult women with AIT have also yielded conflicting results (17,18). The discrepancy in serum AMH levels between our study and these other publications may be due to differences in the participants’ age, number of cases, thyroid status and thyroid disease duration (in the AIT groups), and type of medication used for therapy since thyroxine treatment may relieve all the adverse factors associated with high TSH and thyroid antibodies and restore the ovarian function (19). Some studies have included several patients with polycystic ovary syndrome (PCOS); compared with patients without PCOS, those with PCOS have more antral follicles and, thus, increased secretion of AMH (20).

Our study demonstrated a significant negative correlation between BMI SDS and AMH, which remained significant in regression analysis. This is in agreement with Freeman and cols. (21), who reported the same result, suggesting that folliculogenesis is likely impaired as the BMI increases; indeed, insulin resistance in obese individuals impacts granulosa cells and consequently alters AMH concentration. A lipotoxic effect on the granulosa cells may also be present. Therefore, body weight control may be necessary to preserve ovarian reserve (22).

In the present study, TPOAb levels were significantly higher in patients with overt hypothyroidism than those with euthyroidism or controls. Moreover, the negative correlation of AMH with TPOAb remained significant in regression analysis, suggesting a possible direct relationship between TPOAb and ovarian reserve. Monteleone and cols. (23) demonstrated the presence of antithyroid antibodies in follicular fluid in women with AIT. However, Özalp Akin and Aycan (5) reported no correlation between AMH and TPOAb or TGAb serum levels in a cohort of adolescent girls with euthyroid AIT (on treatment). The TPOAb that passes through the blood follicle barrier during follicular evolution may cause antibody-mediated cytotoxicity in the growing ovarian follicle and damage to the maturing oocyte, resulting in the destruction and damaging of growing follicles and oocytes via thyroid hormone receptors on these cells (24). Autoimmune antibodies, directly and indirectly, impact folliculogenesis via a change in follicular fluid composition and granulosa cell differentiation, as well as abnormal steroidogenesis via the hypothalamic-pituitary-gonadal axis (25,26). Prospective, randomized, and controlled trials are recommended to confirm these results and clarify the role of AMH as a marker of ovarian reserve in adolescent girls with AIT.

We also demonstrated in this study a significant negative correlation between AMH and serum TSH levels, which remained significant in regression analysis, suggesting a possible direct relationship between TSH and ovarian reserve. This is in agreement with the findings by Özalp Akin and Aycan (5), who reported a negative correlation between serum AMH and TSH levels in adolescent girls with AIT. On the other hand, Tuten and cols. (17) reported no correlation between AMH and TSH serum levels in adult patients with AIT. Elevated TSH levels may have harmful effects on ovarian function as they may directly suppress follicle development or influence the reproductive system via thyroid hormone receptors on the surface of oocytes or through disruption of gonadotropin-releasing hormone (GnRH) function due to increased prolactin secretion (4,5). In addition, depleted thyroid hormone secretion may adversely affect follicle recruitment in patients with overt hypothyroidism (27,28). The follicular fluid typically contains measurable FT3 and FT4, which play a significant role in follicle development and oocyte maturation and quality. Impaired thyroid hormone production is involved in the disruption of the hypothalamic-pituitary-gonadal axis, leading to follicle growth disorder (29).

In conclusion, the results of the present study indicate that the ovarian reserve of adolescent girls with AIT, as measured by serum AMH levels, is affected by thyroid autoimmunity and hypothyroidism, indicating a possible need for monitoring ovarian reserve in these patients.
